# Transition to bereavement: A prospective longitudinal study of health-related quality of life in informal caregivers of oldest-old individuals

**DOI:** 10.3389/fmed.2022.1031143

**Published:** 2022-11-30

**Authors:** Sara Alves, Constança Paúl, Oscar Ribeiro

**Affiliations:** ^1^Center for Health Technology and Services Research (CINTESIS@RISE), ICBAS, Porto, Portugal; ^2^Abel Salazar Institute of Biomedical Sciences – University of Porto (ICBAS-UP), Porto, Portugal; ^3^Center for Health Technology and Services Research (CINTESIS.UA), University of Aveiro, Aveiro, Portugal; ^4^Department of Education and Psychology, University of Aveiro, Aveiro, Portugal

**Keywords:** bereavement, caregivers, aged 80 and over, patient care, quality of life, adaptation, psychological, emotional adjustment

## Abstract

**Introduction:**

Experiencing bereavement may be challenging. Despite the oldest-old population increase, a subgroup at greater risk of death, few studies focus on the grieving process of informal caregivers (ICs). This study analyzed the transition to bereavement of ICs of oldest-old individuals (≥80 years) over 1-year and compares the evolution of the health-related quality of life (HrQoL) between those experiencing bereavement and those who continued care through the study period.

**Materials and methods:**

A prospective longitudinal observational study was conducted enrolling 204 ICs of the Metropolitan Area of Porto (North Portugal), of which 36 experienced the death of care receiver (CR). ICs’ health profile and burden were assessed. CRs’ functional and cognitive status were also appraised.

**Results:**

Bereaving caregivers were mostly female, CRs’ children, and had on average 60.4 years at baseline. Caregivers spent a mean of 10.1 h/day (SD = 7.7) caring, for 80.6 months (SD = 57.5). The time elapsed since CR’s death was 6 months (SD = 3.5) from entering in the study. CRs who died had a mean age of 88.3 (SD = 5.4) years at baseline, and were very dependent. Over a 1-year follow-up, bereaving caregivers showed a significant decrease in mental health following CR’s death; on the other hand, caregivers who continued caring improved mental health [*F*(1, 159) = 4.249, *p* = 0.041].

**Discussion:**

Ending the caregiver career was marked by a decline in mental health whereas to continue caring was marked by an improvement in this outcome. While it is highly expected that the CR’s death will be perceived as a relief considering both the caregiver’s characteristics (e.g., medicines) and the CR condition (e.g., high dependence levels), the results suggest an opposite direction. CRs’ death seems to arise an emotional burden for IC, at least during the first year, possibly triggering feelings of loneliness and a life without purpose that seems to aggravate mental health issues.

**Conclusion:**

The transition to bereavement among ICs seems to lead to a caregiver mental health decline while those who continued caring (and thereby, experiencing caregiving stressors) seems to improve in this outcome. Ceasing caregiving stressors does not seem to contribute better experiencing bereavement among ICs, suggesting the need for support throughout this phase.

## Introduction

According to last population census (2021), in Portugal, oldest old (those aged 80 and above) represented approximately 7.0% (713,164) of the total population (10,344,802) and 29.4% of the population aged 65+ (2,424,122) (1). Trends show that living longer may lead to a long period of disability and frailty with increasing care demands and, data suggest care of the oldest old may rely strongly on informal caregivers considering the low proportion of old individuals living in nursing homes (approximately 4.0%) (2). In Portugal, it misses national information about the real number of informal caregivers. According to an estimating study of the prevalence of informal caregiving in Europe among the population aged 50+ years (those that are more prone to care for old individuals), a proportion of 13.0% was found in Portugal (3). Regarding Spain, a country with similar context (e.g., location, culture, familial culture), a proportion of 14.0% was found, which is quite near from that observed in Portugal (3). Notwithstanding, the absence of a clear information of the magnitude of informal care provision in Portugal, it is well acknowledge that caring for oldest old individuals can have a significant impact in the informal caregivers’ physical and mental health status (4–6). Previous research reported that caregivers of oldest-old individuals tend to show higher levels of burden, lower self-perception of health, and weaker social support networks (7). The oldest old are at a greater risk of experiencing negative events such as falls (8), hospitalization (9, 10), and death (11, 12). The influence that such events may have on caregiving course, especially its impact on caregivers’ quality of life, reinforces the need of to be attentive to the dynamics of the caregiving trajectory (13).

Seltzer and Li (14) mentioned that the effects of the caregiving role on the caregivers’ well-being are not static, adding that transitions across the caregiving process may affect caregivers’ quality of life. One of the most widely used conceptual frameworks to examine caregiving trajectories is Pearlin’s Stress Process Model (1990) (15). It highlights the concept of “caregiving career” characterizing informal caregiving as a long-term activity with two main transitions over the time (16): one referring to a change from home care to the institutionalization of the person cared; and another to bereavement due to the death of the person cared. According to these authors, such transitions may lead to a reconfiguration of stressors and of resources, meaning that some of the stressors that caregivers are exposed to can be relieved, but others may be generated requiring from caregivers other or new strategies to face such changes (16).

The current literature on informal caregiving issues (4, 6, 17) – including the study of informal caregiving of oldest-old individuals – tend to focus on describing the negative impacts associated with the experience (e.g., depression, physical and mental burdens). How caregivers may experience grief and bereavement has received significantly less attention. Concretely, the literature on caregivers’ experience of bereavement has been focusing on specific groups (e.g., caregivers of individuals with dementia, of palliative patients) (14, 18–22), leaving the experience of bereaving for oldest old individuals poorly understood. Whereas some studies have documented improvements in the caregivers’ quality of life, such as higher psychological well-being (14), relief (21), lower symptoms of depression and anxiety (21), gratefulness (23, 24) and an overall better social functioning (14) in the bereavement period; others have evidenced a worsening of the caregivers’ quality of life (25) including increased levels of depression (26–28), anxiety (27, 28), lower self-perception of health (19, 28), unresolved regrets (29, 30) and lower engagement in social activities (20, 26, 27) from the period of caregiving to the period of bereavement. Some differences across the results relate to time spent on caregiving (higher length of caregiving was related to a difficult bereavement), relationship to the person being cared for (adult children experienced a stronger form of grief, while the spouses’ grief was found to be quiet and sad), and/or related to the caregivers’ health condition and burden (poorer health condition and higher burden increased caregiver grief). These findings suggest that this period can be particularly demanding for informal caregivers of oldest-old individuals because care provision is usually long-lasting (20, 31), intensive (21, 32), more burdening (5) and the caregiver usually presents a vulnerable health condition (e.g., concomitant diseases, many medicines intake) (4, 17). Besides, high mortality rates are found in oldest-old individuals (33, 34) which probably increases the proportion of informal caregivers experiencing such an event and therefore may also increase the number of individuals at risk.

A deep understanding of how caregivers experience bereavement within the caregiving trajectory is crucial for planning supportive interventions such as anticipatory preparation to care receivers’ death, psychological support, and engagement in social activities. Accordingly, the heterogeneous effects found in bereaved caregivers, showing both losses and improvements in caregivers’ quality of life after the care receivers’ death, stresses the need to have more information about the caregiving trajectory in a comprehensive manner. Rather than considering a specific period of the caregiving trajectory (e.g., only the caregiving period or only the bereavement period), it is utmost importance to analyze the patterns of changes across the caregiving trajectory, i.e., to examine the changes observed from caregiving to bereavement.

Considering the current gap in the literature about the impact of role transitions in informal caregivers of oldest-old individuals, we conducted a 1-year prospective longitudinal study encompassing dyads of oldest individuals, i.e., informal caregivers and their oldest-old care receivers. For this study we posed two main objectives. First, to describe the proportion of caregivers who continued providing care and of those who experienced bereavement over 1-year period. Second, to investigate whether the death of the person being cared for (role transition) occurs in tandem with changes in caregivers’ health-related quality of life.

## Materials and methods

### Design

**FIGURE 1:**
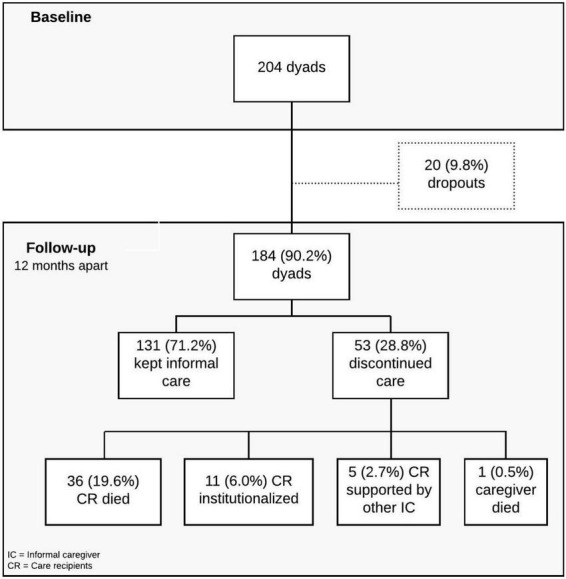
Flowchart of the study.

The data of this study derive from a 1-year prospective longitudinal study designed to explore caregivers and care receivers’ health characteristics and changes in the caregiving context. A non-probabilistic sample of 204 dyads of informal caregivers and oldest-old care receivers were enrolled in the study. Participants were recruited from June 2017 to July 2018 in the Metropolitan Area of Porto (North Portugal), based on the referral by local non-governmental organizations (NGOs) (e.g., day centers and home care services) and on a snowball strategy (35). Dyads were evaluated on two different occasions: at baseline and 12 months later (follow-up). Of the 204 dyads enrolled at the beginning of the study, 184 were reassessed at follow-up (Figure 1), when the following outcomes were observed in the caregiving situation: the caregiver continued providing care; the caregiver ended care provision due the care receiver’s death; the caregiver stopped care provision because the care receiver was institutionalized or because the care provision was delegated to another person; or the caregiver died (Figure 1). This manuscript reports the findings related to the ending of care provision caused by the death of the person in care (Figure 1). Other findings from this study are described elsewhere (17).

Participants were recruited by referral from local non-governmental organizations (NGOs) (e.g., day centers and home care services) and by resorting to a snowball strategy (35). A two-stage process was used: first, local NGOs were invited to participate in the project. Those that accepted to participate in the study identified possible participants according to inclusion criteria: (a) care receiver aged 80+ years; (b) having a designated informal caregiver; (c) living in the community; and (e) residing in the Metropolitan Area of Porto. The secretary of each organization contacted each potential participant to ask for authorization for sharing personal data with the research team. After this preliminary consent, the research team contacted the potential participants and provided a more detailed description of the study, namely its objectives and conditions. For those who accepted to participate, a face-to-face interview was scheduled and both caregivers and care receivers answered to the questions comprising the assessment protocol. All participants were interviewed by a researcher (at that time also PhD student), with experience on informal caregiving issues and aging as well as on data collection. Each interview took approximately 60–90 min to 30–45 min for the informal caregiver and, 30–45 min for the care receiver. If the oldest old person had no cognitive ability to respond (e.g., people with dementia), permission to participate was obtained from the legal representative, which in most cases coincided with the informal caregiver. All participants signed an informed consent form.

One year apart from the baseline assessment, participants were contacted by phone to know about the caregiving situation and willingness to participate in a follow-up assessment. For those who continued care and agreed to continue in the study, a face-to-face interview was scheduled and the caregiving dyad was reassessed with the same assessment protocol used at baseline; for those who were no longer providing care, a brief phone interview including information on their health status and the motives for the change of the caregiving situation (e.g., death and institutionalization) was conducted. The study was approved by the Ethical Committee of the Institute of Biomedical Sciences of Abel Salazar, University of Porto (process no. 188/2017) and authorized by the Portuguese Data Protection Authority (approval no. 1338/2017).

### Measures

At baseline, caregivers and the care receivers answered the questions in the following assessment protocol:

– Dyad’s sociodemographic and caregiving context information: Personal data (e.g., age, sex, relationship with care receivers) and information about the caregiving context (e.g., co-residence, caregiving time and duration).
– Informal caregiver health status and caregiving outcomes:
Health status: (a) number of medicines used; (b) number of diseases; (c) health-related quality of life, as measured by the Portuguese version of the MOS Short-Form 12 Health Survey (SF – 12v2) (36–38). This instrument has 12 items distributed by two main component scores: physical and mental health. The Physical Component Summary (PCS) and Mental Component Summary (MCS) scores were calculated using weighted item composites. These scores are standardized (*T* scores) to have population means of 50 and standard deviations of 10, with higher scores reflecting better functioning. The final score of PCS ranges from 11.14 to 69.35 and MCS ranges from 7.35 to 73.15. Despite these two-component summary dimensions, it is also possible to obtain scores about eight health domain scales, namely: (i) Physical Functioning, which evaluates the performance in physical activities and ranges from 25.58 to 57.06 points; (ii) Role Physical, which assesses problems in accomplishing as much work or other daily activities as one would like and ranges from 23.61 to 57.46 points; (iii) Bodily Pain, which evaluates the interference of pain with normal work/activities and ranges from 21.66 to 57.73 points; (iv) General Health, which evaluates one’s perception of health and range from 23.90 to 63.66 points, (v) Vitality, which assesses the energy of the person and ranges from 29.39 to 68.74 points; (vi) Social Functioning, which evaluates the interference of physical and emotional problems with social activities and ranges from 21.32 to 56.90 points; (vii) Role Emotional, which assesses experiencing problems in accomplishing as much work or other daily activities as one would like and ranges from 14.70 to 56.28 points; and (viii) Mental Health, which evaluates feeling downhearted and depressed and ranges from 18.32 to 64.21 points.
Caregiving burden. This caregiving outcome was assessed by the screening version of the Zarit Burden Interview (39, 40). This instrument comprises four questions evaluated in a 5-point Likert Scale, from 0 (never) to 4 (nearly always). The final score ranges from 0 to 16 points and the higher the score, the higher the overload of the informal caregiver; scores of ≥ 7 indicate the presence of high burden. A Cronbach’s alpha of 0.71 were found in the Portuguese version of ZBI-4 (39).
– Care receiver functional and health status:
Basic activities of daily living. We assessed care receivers’ level of dependence through the Barthel Index (41). The final score in the Portuguese version ranges from 0 to 20 points, being that the lower the scores, the higher the level of dependence (42); The Portuguese version (42) has good psychometric properties including optimal internal consistency (Cronbach’s alpha = 0.96).
Instrumental activities of daily living. We assessed the level of dependence of care receivers in IADLs by using Lawton and Brody Scales (43, 44). The final score ranges from 8 to 30 points, being that the lower the scores, the better the performance. The Portuguese version of the scale showed a Cronbach’s alpha of 0.94.
Cognitive status: The Mini-Mental State Examination (45, 46) was used to assess the cognitive performance of care receivers. The final score ranges from 0 to 30 points and the lower the scores, the worst the cognitive performance. Good psychometric properties (46) were found in the Portuguese version, including a Cronbach’s alpha of 0.89.

At follow-up, those who continued providing care were reassessed with the same evaluation protocol used at baseline (except the dyad’s sociodemographic data). Those who experienced the death of the person cared answered only answered questions about their health status (same questions as at baseline assessment: see informal caregiver health status above).

### Statistical analysis

The characteristics of the sample were summarized using IBM SPSS v.25 (SPSS Inc., Chicago, IL, USA) for descriptive statistical analysis. Results were presented in absolute and relative frequencies or central location and dispersion measures, depending on the type of variable. To ensure that the results of this study were not affected by attrition, we compared those who completed the study with dropout cases (9.8%) across the sociodemographic, health, and care context variables of the dyads. We used the Mann–Whitney *U* test for the continuous variables as assumptions of normality for running parametric tests were not met, and the Chi-Square test or the Fisher’s exact test for the categorical variables. The analysis showed that those who dropped out from the study did not differ significantly from those who completed the study. Additionally, both groups were compared for sociodemographic, health and context characteristics at baseline, aiming to detect differences between the two groups. We used independent *t*-tests to compare continuous sociodemographic, context, and health variables (e.g., age and length of care provision). To perform the comparison for categorical variables (e.g., sex and co-residence), Chi-Square test or Fisher’s exact tests were used, following the same procedure for the comparison of the groups. Appropriate central tendency and dispersion measures as well as relative and absolute frequencies were used to describe the data (see Table 1).

**TABLE 1:** Descriptive analysis for sociodemographic, context, and health characteristics’ of participants and differences between groups, at baseline.

	Groups		
	Kept informal care	Ended informal care (Bereavement)	*p*
	*n* = 131	*n* = 36	
**Informal caregiver**			
Age (mean, SD)	59.4 (9.3)	60.4 (9.1)	0.576
Female sex, *n* (%)	108 (83.2)	32 (88.9)	0.604^a^
Education level, *n* (%)			0.638
≤4 years	55 (42.0)	12 (33.3)	
5–9 years	36 (27.5)	11 (30.6)	
≥10 years	40 (30.5)	13 (36.1)	
Co-residence, *n* (%)			0.843^a^
Yes	86 (66.4)	23 (63.9)	
No	44 (33.6)	13 (36.1)	
Relationship with care receivers, *n* (%)			0.883
Spouse/husband/partner	8 (6.1)	3 (8.3)	
Children	101 (77.9)	27 (75.0)	
Other	21 (16.0)	6 (16.7)	
Caregiving time (mean hours/day, SD)	9.6 (7.8)	10.1 (7.7)	0.738
Caregiving length in months, mean (SD)	84.0 (72.7)	80.6 (57.5)	0.791
No. medicines intake, mean (SD)	2.4 (2.3)	2.9 (3.1)	0.242
No. of diseases, mean (SD)	4.5 (2.4)	4.4 (2.4)	0.707
ZBI-4 total score, mean (SD)	6.2 (4.0)	6.1 (3.9)	0.887
Length of grief, months (SD)	–	5.9 (3.5)	–
**Care receiver**			
Age (mean, SD)	87.7 (4.9)	88.3 (5.4)	0.068
Female sex, *n* (%)	102 (78.6)	30 (83.3)	0.644^a^
ADL, mean (SD)	10.3 (6.6)	7.9 (7.0)	0.100
IADL, mean (SD)	26.6 (3.7)	26.8 (3.8)	0.819
MMSE, mean (SD)	16.4 (8.7)	13.6 (10.9)	0.171

^a^Fisher’s exact test.

For the second aim of this study, we conducted two-way repeated-measures analyses of variance (ANOVA). The repeated measures were taken at baseline and follow-up (1 year apart) and reflected caregivers’ health-related quality of life. From the total of individuals enrolled in this study (*N* = 167), only 161 entered this analysis due to missing values on this outcome (SF12v2). The two-way repeated-measures ANOVAs tested three types of effects: the main effect of caregiving status/groups (kept or end of informal care provision), the main effect of time, and the interaction of caregivers’ status/groups × time. Our greatest interest was in the interaction effect, which indicated whether there was a pattern of change from baseline to follow-up manifested by those who experienced a transition in caregiving status as compared with those who did not change the caregiving status. We refer to this as a caregiving transition “effect,” although causal conclusions are not warranted based on this research design. The validity of the comparisons was strengthen considering the previous analysis showing that these groups did not differ at baseline, and so there was no need to adjust for sociodemographic, context, and health variables in ANOVAs analyses. A significance level of α = 0.05 was considered for all the analyses.

## Results

### The transition of the caregiving situation

Following Figure 1, 71.2% of participants kept informal care provision and 28.8% discontinued the provision of care 1 year apart from baseline. From those who discontinued care provision, 19.6% of caregivers experienced the death of the person being cared for, 6.0% experienced the institutionalization of the care receiver, 2.7% delegated care provision to another informal caregiver, and one caregiver died (0.5%).

### The characteristics of the study participants

Results of sociodemographic, context of care and health information for each group (informal caregivers that continued providing care vs. bereaved caregivers) are presented in Table 1. Overall, the groups did not differ significantly for any of the sociodemographic, caregiving context, and health characteristics at baseline, suggesting that the two groups were quite similar.

Informal caregivers that continued providing informal care.

Participants (*N* = 131) had an average age of 59.4 years, were mostly women, offspring, with low educational level, and lived with the person they care. The mean time of care was 9.6 h/day and the caregiving length was 84.0 months, i.e., approximately 7 years. Caregivers presented a mean of 2.4 medicines intake, a mean of 4.5 diseases, and intermediate levels of caregiving burden (6.2 points). As for the care receivers, they had a mean age of 87.7 years, were mostly females, and presented high levels of disability both for BADLs and IADLs, and a mean of 16.4 points in the MMSE.

Bereaved caregivers.

The mean age of this group (*N* = 36) was 60.4 years. The majority of the sample was female, children, and lived with the care receiver. This group evidenced a similar distribution across the educational level groups’ (33.3, 30.6, and 36.1% for ≤ 4, 5–9, ≥ 10 of schooling years, respectively). Regarding the length of grief, it was on average 6 months. Considering the caregiving context, the average time spent on care was 10.1 h/day and the average length of care provision was 80.6 months, i.e., approximately 6.7 years. For the health and caregiving outcomes, results revealed a mean of 2.9 medicines intake, 4.4 diseases, and 6.1 points in caregiving burden. Concerning care receivers’ characteristics, most were women, with a mean age of 88.3 years, and presented high levels of dependency. The MMSE scores revealed a mean of 13.6 points.

### Changes in health-related quality of life

Table 2 contrasted the two groups of caregivers at baseline and follow-up (1 year apart) and one significant result emerged. Concretely, an interaction effect of Caregivers’ status/groups × Time (i.e., bereavement effect) was observed: bereaved caregivers decreased in mental health summary scores *F*(1, 159) = 4.249, *p* < 0.05 following the death of the care receiver, whereas caregivers who kept in the caregiving role increased in this dimension during the study period.

**TABLE 2:** Comparison of health-related quality of life between the two groups.

				Caregivers’ status/Groups				Time				Caregivers’ status/Groups × Time			
Measure/ Scale		Baseline M (SD)	Follow-up M (SD)	df	*F*	*p*	Effect size	df	*F*	*p*	Effect size	df	*F*	*p*	Effect size
Physical Component	Kept (*n* = 128)	47.0 (8.7)	47.6 (8.8)	(1, 159)	2.34	0.128	0.014	(1, 159)	0.762	0.384	0.005	(1, 159)	0.025	0.876	0.000
Summary (PCS)	Ended (*n* = 33)	49.1 (9.7)	50.0 (7.3)												
Mental Component	Kept (*n* = 128)	44.7 (12.1)	47.4 (12.6)	(1, 159)	0.497	0.482	0.003	(1, 159)	0.014	0.906	0.000	(1, 159)	4.249	0.041*	**0.026^†^**
Summary (MCS)	Ended (*n* = 33)	45.7 (13.5)	43.3 (13.5)												
Subscales															
Physical functioning	Kept (*n* = 128)	48.2 (9.8)	47.5 (10.8)	(1, 159)	0.000	0.996	0.000	(1, 159)	0.800	0.372	0.005	(1, 159)	0.000	1.000	0.000
	Ended (*n* = 33)	48.2 (5.7)	47.5 (9.4)												
Role Functioning	Kept (*n* = 128)	47.1 (11.0)	48.0 (11.4)	(1, 159)	0.523	0.471	0.003	(1, 159)	1.189	0.277	0.007	(1, 159)	0.235	0.628	0.001
	Ended (*n* = 33)	48.0 (10.7)	49.8 (10.5)												
Bodily pain	Kept (*n* = 128)	49.0 (11.1)	51.1 (10.6)	(1, 159)	0.004	0.949	0.000	(1, 159)	1.206	0.274	0.008	(1, 159)	0.235	0.628	0.001
	Ended (*n* = 33)	49.5 (13.2)	50.4 (10.4)												
General health	Kept (*n* = 128)	40.1 (11.0)	39.3 (11.6)	(1, 159)	3.517	0.063	0.022	(1, 159)	0.427	0.514	0.003	(1, 159)	2.269	0.134	0.014
	Ended (*n* = 33)	42.6 (12.3)	44.5 (11.2)												
Vitality	Kept (*n* = 127)^a^	47.2 (11.0)	49.5 (11.9)	(1, 158)	1.613	0.206	0.010	(1, 158)	0.039	0.844	0.000	(1, 158)	2.290	0.132	0.014
	Ended (*n* = 33)	47.0 (12.5)	45.2 (11.0)												
Social functioning	Kept (*n* = 128)	49.3 (11.7)	50.0 (12.0)	(1, 159)	0.006	0.937	0.000	(1, 159)	0.681	0.411	0.004	(1, 159)	1.953	0.164	0.012
	Ended (*n* = 33)	51.0 (11.9)	48.0 (12.8)												
Role emotional	Kept (*n* = 127)^a^	45.8 (11.7)	47.5 (12.0)	(1, 158)	0.643	0.424	0.004	(1, 158)	0.020	0.887	0.000	(1, 158)	2.655	0.105	0.017
	Ended (*n* = 33)	46.0 (12.5)	44.0 (12.9)												
Mental health	Kept (*n* = 128)	42.1 (14.0)	44.9 (13.3)	(1, 159)	0.041	0.841	0.000	(1, 159)	0.777	0.379	0.005	(1, 159)	1.640	0.202	0.010
	Ended (*n* = 33)	44.2 (13.9)	43.7 (15.1)												

^a^For these subscales, the *N* = 127 due to missing information. *p < 0.05. ^†^According to Cohen’s criteria: low size effect. Bold values represent the *p* < 0.05.

## Discussion

This study aimed to explore a sample of informal caregivers of oldest-old individuals contrasting those who experienced bereavement and those who continue providing care over 1-year for health-related quality of life. Also, we aimed to understand if the transition to bereavement occurs with changes in caregivers’ health-related quality of life. From the findings, two main aspects should be highlighted: first, the proportion of individuals experiencing bereavement; and second, the different evolution of caregivers’ mental health observed over 1 year in those that continue providing informal care vs. those experiencing bereavement.

Considering the first aspect, the results of this study emphasize the changes of the caregiving situation even in a short period of follow-up. Within 1 year, approximately one-fifth of those who were caregivers at the beginning of the study ended their role due to the death of the care receiver, evidencing a high proportion of individuals experiencing bereavement. Due to the advanced age of care receivers who have a greater risk of mortality (12), this result could be somewhat expected.

As for the second aspect to highlight, our results showed that ending the caregiver career is marked by a decline in mental health whereas to continue providing informal care is marked by an improvement in this outcome. The dynamism of the caregiving trajectory and the effects that emerge thorough time and from caregiving transitions is consistent with Pearlin’s conceptualization (47) and with the definition of caregiving as a career (16). According to the results, the demands of the caregiving role seem to change over time, as well as the effects of the changes on caregivers which could be possibly related to a reconfiguration of stressors (16, 47) which means that some of the stressors that caregivers are exposed can be relieved but others may emerge (22). Caregivers may experience relief, gratefulness and an overall better social functioning (14, 23, 24) or by the contrary, to evidence an increase in the levels of depression and anxiety, lower self-perception of health, lower social interaction unresolved regrets (19, 20, 24, 29). Several studies have documented that the care provision to individuals in advanced age is particularly complex and challenging for the caregiver (5, 6, 17). In fact, in this study, despite the advanced age of care receivers and their high dependency levels (which could increase the caregivers’ awareness about the care receivers’ end-of-life, enabling progressive grief process), informal caregivers seem to present more difficulties to deal with the loss of the person they cared for than to continue providing care, even when the levels of dependence of care receivers increases (as seen in this study). Even considering that the death of a loved one (whom one is caring for or not) often leads to a decrease in mental health, this study suggests that what could be understood as a potential relief regarding caregivers’ health status and the receivers’ high levels of dependence did not happen. According to the results, experiencing the death of the person cared for suggests a worse impact on the caregivers’ mental health condition than continuing to provide care in a circumstance of increased levels of dependence and high levels of burden. Furthermore, it may suggest that caregivers cope better with a challenging caregiving (role adaptation) than with the death of the person being cared for despite the cessation of caregiving stressors. Considering the exploratory nature of this study, it will be interesting to further analyze which factors/determinants enable or hamper this process (e.g., socioeconomic status, burden, health condition, kinship with care receiver). Moreover, it would be important for the study of caregiving trajectories to also consider the trajectory of the post-caregiving period, analyzing at what point time in this trajectory caregivers will have to cope with challenges of grief and bereavement. For instance, in some studies enrolling caregivers of dementia patients it has been observed that caregivers might experience negative consequences of care receivers’ death for 1–2 years (48, 49). Additionally, a recent systematic review analyzing the trajectories of depressive symptoms for bereaved family members of chronically ill patients found that most of the bereaved families endured their grief and adjusted, returning to pre-bereavement depressive-symptom levels within 1-year post-loss (50). In our sample, caregivers were assessed on average 6 months (SD = 3.5) after the loss. This, according to previous studies, may correspond to a period of high vulnerability after the care receivers’ death wherein participants did not have enough time to adjust to the loss of the person and to return to pre-bereavement mental health levels (50). Notwithstanding, this result should be further explored once it may suggest increased susceptibility of informal caregivers of oldest-old in the bereavement period, reinforcing the need of policy and practical amendments to higher support across the grief process. Also, the comparison between the two groups for sociodemographic, context and health variables revealed that both groups are quite similar strengthening the probable effect of bereavement in caregivers’ mental health. It is interesting to note that those who did not experience a role transition – keep providing informal care – improved their mental health suggesting a caregiver continuous adaptation over the caregiving trajectory (16, 51, 52). In fact, the interaction effects observed between the caregivers’ status/groups and time in mental health could indicate that it is more difficult to experience the transition for grief than the continuity of informal care provision.

Some limitations are to be mentioned. Firstly, the evidence found in this 1-year study should be further studied. It would be interesting to conduct a longer longitudinal study to evaluate informal caregiving since the beginning to the end of the caregiving trajectory, following the role transitions and evaluating the caregivers’ outcomes over ageing and illness trajectories. Understanding the changes that emerge throughout time, and how they impact the caregivers’ health could inform the design of interventions in the caregiving continuum at such advanced ages (7). As the sample size of those experiencing bereavement did not allow to further explore the results across groups (for instance, considering the kinship with the care receiver), it would also be relevant to understand how the caregiving trajectory and the role transitions are experienced by spouses (probably with advanced ages themselves) vs. offspring. Likewise, it would be insightful to re-assess this sample of bereaved caregivers later in time to verify if the decline in mental health persists or if it returns to pre-bereavement levels. Other limitation regards to the lack of representativeness of the sample and the small sample size (particularly of those who experienced bereavement), limiting the generalizability of the results. Thereby, the findings from this study should be seen as hypothesis-generating and propel further research. Despite the study limitations, it is important to stress that the findings obtained in this study are to some extent worrisome – even though no causal relationship could be derived, the observed decline in the caregivers’ mental health after the care receivers’ death highlights the need for better monitoring the post-caregiving period and recognizing it as a phase of the caregiving career, as suggested by several researchers (18, 53). This aspect reinforces the need for interventions to support bereavement, alleviating the grief and the continuing of caregivers’ well-being, self-directedness, social engagement, and participation in meaningful activities (22, 54).

Lastly, our results may have some policy and practical implications that could generally be addressed (22). Overall, these results may offer future directions in terms of policy (e.g., to accurately plan services and to manage staff requirements) and practice (e.g., to proper define the frequency of dyads monitoring, to intervene or referrer for intervention individuals at greater risk of death). Some policies may include for instance more resources targeting home-based care, helping caregivers to prepare for the grief and bereavement period; and the increasing of bereavement leaves, and/or to regulate mandatory appointments through primary health care to closely monitor caregivers during this challenging period. Practical implications may consider the offering of education and training on the differences in roles and grief experiences of family members, increasing the training of professionals for bereavement support including not physical, psychosocial, and spiritual care but also enabling them to prepare caregivers to experience bereavement during end-of-life discussions, and/or peer support groups like mutual aid groups.

This study aimed to contribute to the increase of knowledge on bereavement among informal caregivers of oldest old individuals. The understanding of the factors that interfere in caregivers’ quality of life and specifically mental health represents a big challenge considering the high number of aspects that can affect the normal functioning of caregivers’ mental health and whose effects usually overcome the caregiving period.

## Data availability statement

The raw data supporting the conclusions of this article will be made available by the authors, without undue reservation.

## Ethics statement

This study was approved by the Ethical Committee of the Institute of Biomedical Sciences of Abel Salazar, University of Porto (process no. 188/2017) and authorized by the Portuguese Data Protection Authority (approval no. 1338/2017), guaranteeing anonymity, privacy and confidentiality. The patients/participants provided their written informed consent to participate in this study.

## Author contributions

SA was responsible for the study design, collecting, analyzing and interpreted data, article drafting and revision. CP and OR was responsible for study supervision and article revision. All authors contributed to the article and approved the submitted version.
